# Dual inhibition of SUMOylation and MEK conquers MYC-expressing *KRAS*-mutant cancers by accumulating DNA damage

**DOI:** 10.1186/s12929-024-01060-3

**Published:** 2024-07-11

**Authors:** Hiroshi Kotani, Hiroko Oshima, Justin C. Boucher, Tomoyoshi Yamano, Hiroyuki Sakaguchi, Shigeki Sato, Koji Fukuda, Akihiro Nishiyama, Kaname Yamashita, Koushiro Ohtsubo, Shinji Takeuchi, Takumi Nishiuchi, Masanobu Oshima, Marco L. Davila, Seiji Yano

**Affiliations:** 1 Division of Medical Oncology, Cancer Research Institute, Kanazawa University, 13-1 Takara-Machi, Kanazawa, Ishikawa 920-0934 Japan; 2 Division of Genetics, Cancer Research Institute, Kanazawa University, Kanazawa, Japan; 3 Nano Life Science Institute, Kanazawa University, Kanazawa, Japan; 4 Department of Blood and Marrow Transplant and Cellular Immunotherapy, Division of Clinical Science, H. Lee Moffitt Cancer Center, Tampa, FL USA; 5 Department of Immunology, Graduate School of Medical Sciences, Kanazawa University, Kanazawa, Japan; 6 Research Center for Experimental Modeling of Human Disease, Kanazawa University, Kanazawa, Japan; 7 Department of Medicine, Roswell Park Comprehensive Cancer Center, Buffalo, NY USA; 8 Department of Respiratory Medicine, Graduate School of Medical Sciences, Kanazawa University, Kanazawa, Japan

**Keywords:** KRAS, MYC, SUMOylation, MEK, DNA damage

## Abstract

**Background:**

*KRAS* mutations frequently occur in cancers, particularly pancreatic ductal adenocarcinoma, colorectal cancer, and non-small cell lung cancer. Although *KRAS*^*G12C*^ inhibitors have recently been approved, effective precision therapies have not yet been established for all *KRAS*-mutant cancers. Many treatments for *KRAS*-mutant cancers, including epigenome-targeted drugs, are currently under investigation. Small ubiquitin-like modifier (SUMO) proteins are a family of small proteins covalently attached to and detached from other proteins in cells via the processes called SUMOylation and de-SUMOylation. We assessed whether SUMOylation inhibition was effective in *KRAS*-mutant cancer cells.

**Methods:**

The efficacy of the first-in-class SUMO-activating enzyme E inhibitor TAK-981 (subasumstat) was assessed in multiple human and mouse *KRAS*-mutated cancer cell lines. A gene expression assay using a TaqMan array was used to identify biomarkers of TAK-981 efficacy. The biological roles of SUMOylation inhibition and subsequent regulatory mechanisms were investigated using immunoblot analysis, immunofluorescence assays, and mouse models.

**Results:**

We discovered that TAK-981 downregulated the expression of the currently undruggable MYC and effectively suppressed the growth of MYC-expressing *KRAS*-mutant cancers across different tissue types. Moreover, TAK-981-resistant cells were sensitized to SUMOylation inhibition via MYC-overexpression. TAK-981 induced proteasomal degradation of MYC by altering the balance between SUMOylation and ubiquitination and promoting the binding of MYC and Fbxw7, a key factor in the ubiquitin–proteasome system. The efficacy of TAK-981 monotherapy in immunocompetent and immunodeficient mouse models using a mouse-derived CMT167 cell line was significant but modest. Since MAPK inhibition of the KRAS downstream pathway is crucial in *KRAS*-mutant cancer, we expected that co-inhibition of SUMOylation and MEK might be a good option. Surprisingly, combination treatment with TAK-981 and trametinib dramatically induced apoptosis in multiple cell lines and gene-engineered mouse-derived organoids. Moreover, combination therapy resulted in long-term tumor regression in mouse models using cell lines of different tissue types. Finally, we revealed that combination therapy complementally inhibited Rad51 and BRCA1 and accumulated DNA damage.

**Conclusions:**

We found that MYC downregulation occurred via SUMOylation inhibition in *KRAS*-mutant cancer cells. Our findings indicate that dual inhibition of SUMOylation and MEK may be a promising treatment for MYC-expressing *KRAS*-mutant cancers by enhancing DNA damage accumulation.

**Supplementary Information:**

The online version contains supplementary material available at 10.1186/s12929-024-01060-3.

## Introduction

*KRAS* is the most frequently mutated driver oncogene in ~ 20% of all types of cancer. In particular, the frequencies of *KRAS* mutations are high in pancreatic ductal adenocarcinoma (PDAC; ~ 90%), colorectal cancer (CRC; ~ 45%), and non-small cell lung cancer (NSCLC; ~ 30%) [1]. Although *KRAS*^*G12C*^-specific inhibitors, such as sotorasib and adagrasib, have been encouraged in recent years because of the success of drugging ‘undruggable’ targets [2–4], various treatment approaches tested for *KRAS*-mutant cancers, excluding NSCLC with the *KRAS*^*G12C*^ mutation, have not achieved clinical success. Given the unmet medical needs of patients with other *KRAS* mutations or primary/acquired resistance to *KRAS*^*G12C*^ inhibitors, novel therapeutic approaches should be considered [5, 6].

A potential treatment for suppressing *KRAS*-mutant cancer progression is blocking the small ubiquitin-like modifier (SUMO) pathway [7, 8]. SUMOs are post-translational modifications (PTMs) that regulate a wide variety of proteins in many pathways. The conjugation of SUMO proteins to substrate proteins is called SUMOylation. An enzymatic cascade consisting of dimeric SUMO-activating enzyme E1 (SAE1/SAE2), a single E2 ubiquitin-conjugating enzyme 9 (UBC9), and a limited set of E3 ligases causes SUMOylation [9, 10]. SUMOylated substrates can alter protein–protein interactions, change protein intracellular localization, or directly change protein activity. Functional SUMO family members in mammals mainly consist of SUMO1, SUMO2, and SUMO3. SUMO2 and SUMO3 are frequently referred to as SUMO2/3 because they share a 97% sequence, whereas SUMO1 shares less than a 50% sequence with SUMO2/3. Additionally, SUMOylated substrates can reversibly deconjugate by SUMO-specific proteases (SENPs). Recently, a first-in-class, highly selective small-molecule SAE inhibitor, TAK-981 (subasumstat), which leads to SUMOylation inhibition, was developed as an immunomodulating drug [11–13]. While it is being tested in clinical trials for solid tumors and hematological malignancies (NCT03648372, NCT04074330, NCT04776018, and NCT04381650), SUMOylation inhibition potentially reduces cancer cell proliferation [14]. Therefore, we expected that SUMOylation inhibition might be beneficial for treating *KRAS*-mutant cancers.

Here, we investigated the sensitivity of *KRAS*-mutant cancers to SUMOylation inhibition by TAK-981 and identified a potential predictive biomarker. Moreover, we revealed that co-inhibition of SUMOylation and MEK could conquer *KRAS*-mutant cancers.

## Materials and methods

### Reagents

TAK-981, TAK-243, trametinib, A-83–01, CHIR-99021, Y-27632, and RS-1 were purchased from Selleck Chemicals. 16% paraformaldehyde was purchased from FUJIFILM. Triton X-100 was purchased from Sigma-Aldrich. HEPES, RPMI 1640, DMEM, advanced DMEM/F12, fetal bovine serum, and penicillin/streptomycin were purchased from Gibco.

### Cells

The cell lines and culture conditions are summarized in Supplementary Table 1. AKTP 1C9 and 2A6 cells established by the Oshima lab (Kanazawa University) were cultured in advanced F12/DMEM supplemented with 10% heat-inactivated fetal bovine serum, 100 U/mL penicillin, 100 µg/mL streptomycin, 5 µM A-83–01, 5 µM CHIR-99021, and 10 µM Y-27632. The cells were regularly screened for mycoplasma contamination using the MycoAlert Mycoplasma Detection Kit (Lonza). The cell number and viability were determined using a Countess II FL Automated Cell Counter (Thermo Fisher Scientific). The controls were treated with 0.1% DMSO throughout the experiment, as described in our previous reports [15, 16].

### Immunoblot analysis

Cell lysates were collected using CelLytic M (Sigma-Aldrich) supplemented with 1% phosphatase inhibitor cocktail 3 (Sigma-Aldrich) and 10 µM phenylmethanesulfonyl fluoride (Sigma-Aldrich), and immunodetection of the proteins was performed using standard protocols. The procedures of SUMOylation assay of SAE2 and ubiquitination detection of MYC were performed using a SUMOylation assay kit (Abcam) and a Signal-Seeker Ubiquitination Detection kit (Cytoskeleton, Inc.) according to the manufacturer’s instructions, respectively. For the immunoprecipitation assay, each cell lysate of 1000 µg was incubated with MYC antibody and rmp Protein A Sepharose Fast Flow beads (Cytiva) under gentle rotation. After overnight incubation, the samples were washed thrice and prepared for immunoblotting. Only for immunoprecipitated samples, Clean-Blot IP Detection Reagent (Thermo Fisher Scientific) was used for secondary antibody to reduce background noise. Signals were detected using a Chemiluminescence Imaging System (M&S Instruments Inc.). The antibodies used are listed in Supplementary Table 2.

### Cell viability assay for IC_50_ determination

Cells were seeded in 96-well plates so that the control cells reached approximately 80% confluency at the end of the assay. The next day, cells excluding the control were treated with nine different concentrations of TAK-981 by threefold serial dilution from 10 µM (*n =* 6 at each concentration). After 72 h, cell counting kit-8 (CCK-8) (Dojindo) was added to the cells, and cell viability was determined by measuring the absorbance. IC_50_ values based on viability were calculated using GraphPad Prism 9.

### Gene expression analysis

RNA was extracted using a RNeasy Plus Kit (QIAGEN), and cDNA was synthesized using a SuperScript VILO cDNA Synthesis Kit (Invitrogen) according to the manufacturer’s instructions. A TaqMan Array Human Molecular Mechanisms of Cancer (Applied Biosystems) was used for array analysis in triplicate. The TaqMan Gene Expression Assay was performed in triplicate for *MYC* mRNA expression relative to that in normal cell lines. The amount of amplicon was determined using the Mx3005P qPCR System with TaqMan Universal PCR Master Mix (Applied Biosystems). The expression of each sample was normalized to that of the housekeeping gene *GAPDH*. *MYC* and *GAPDH* were assessed using Hs00153408_m1 and Hs99999905_m1, respectively. The data were analyzed using GraphPad Prism 9.

### Plasmids

GFP and MYC expression plasmids were obtained from Horizon Discovery. Plasmid DNA was amplified according to the instructions and extracted using QIAGEN Plasmid Maxi Kit (QIAGEN). Purified plasmid DNA was transfected into cells using Lipofectamine 3000 Transfection Reagent (Thermo Fisher Scientific). Antibiotic selection was initiated the following day. The antibiotics-containing medium was changed every 2–3 days for two weeks to establish stable cells. The cells were then cultured without antibiotics for at least two days and used for subsequent analyses.

### Trametinib screening data

Drug screening data for trametinib and the mutational status of cell lines were obtained from the Genomics of Drug Sensitivity in Cancer Project (http://www.cancerrxgene.org/).

### Diff-Quik assay

Cells were seeded in 24-well plates so that the control cells would reach 80–100% confluency at the end of the assay. The next day, the cells were treated with 30 nM trametinib, 1 µM TAK-981, or both. After 72 h, the cells were washed twice with PBS, fixed, and stained with Diff-Quik (Sysmex) according to the manufacturer’s instructions.

### Spheroid culture assay

For protein analysis, AKTP 1C9 and 2A6 cells were seeded in 24-well ultra-low attachment plates. After overnight incubation, the cells were treated with the indicated drug(s) for 24 h. For the growth assay, 2 × 10^4^ AKTP 1C9 cells per well were seeded in PrimeSurface 96U plates (FUJIFILM). After overnight incubation, the absorbance of the cells treated with CCK-8 was measured as the baseline, and the remaining cells were treated as described above. The cells were morphologically evaluated five days later using ECLIPSE Ti2 (Nikon). The absorbance of cells treated with CCK-8 was measured after the same incubation duration as that of the baseline control, as in our previous report [16].

### Immunofluorescence staining

Cells that were cultured in a Slide-Chamber (FUKAEKASEI) overnight were treated under specified conditions for 24 h. Cell staining was performed according to the DNA Damage Detection Kit protocol (Dojindo). Briefly, the cells were fixed and permeabilized with 4% paraformaldehyde and 0.1% Triton-X-100 in 250 mM HEPES for 5 min at room temperature (RT) and then treated with 1% Triton X-100 for 20 min at RT. Subsequently, the cells were treated with Blocking Solution for 20 min at RT, stained with an anti-ɣ-H2AX antibody for 1 h at RT, and then stained with a fluorescently labeled secondary antibody for 1 h at RT. Finally, the cells were stained with DAPI and then covered with a micro cover glass (Matsunami Glass). Adequate washing with PBS was performed at each step. Cell images were analyzed using a ZOE Fluorescent Cell Imager (Bio-Rad).

### Mouse models

Female 6- to 8-week-old C57BL/6 J mice (B6) (Charles River Laboratories Japan) and CAnN.Cg-*Foxn1*^*nu*^/CrlCrlj mice (nude) (Charles River Laboratories Japan) were used for this study. The care and treatment of the experimental animals followed institutional guidelines. For immunocompetent or immunodeficient models using mouse-derived cell lines, 1 × 10^6^ CMT167 cells in 100 µL of PBS were subcutaneously injected into the flank of B6 or nude mice on Day -3. For NCI-H2122 xenografts, 2 × 10^6^ cells in 100 µL of growth-factor-reduced Matrigel (Corning)/PBS (50% final concentration) were subcutaneously injected into the flank of nude mice on Day -7. For HCT 116 or MIA PaCa-2 xenografts, 5 × 10^6^ cells or 2 × 10^6^ cells in 100 µL of MatriMix511 (NIP)/PBS (50% final concentration) were subcutaneously injected into the flank of nude mice on Day -7 or -21, respectively. On Day 0, treatment with vehicle (control), trametinib (0.6 mg/kg, oral gavage, daily), TAK-981 (25 mg/kg, intraperitoneal injection, twice/week), or both drugs in combination at the same dose was started after randomization. Trametinib was dissolved in 7% DMSO, 13% Tween 80, 80% glucose, and 6N HCl at an equivalent molar concentration to the drug. TAK-981 was dissolved in 20% HPbCD, 2.5% 1N HCl, 2.25% 1N NaOH, and 75.25% deionized water. The mice were monitored daily for body weight and general condition. Tumor volume was measured twice a week using calipers and was calculated using the following formula: length × width^2^ × 0.5. According to institutional guidelines, mice were sacrificed when their tumor volume reached 1,000 mm^3^. The relative tumor volume (RTV) was calculated using the following formula: RTV = (tumor volume on the measured day)/(tumor volume on day 0).

### Statistical analysis

The group size was determined based on preliminary experimental results and no statistical method was used to predetermine the sample size. The indicated sample sizes (n) represent the biological replicates. Statistical significance was determined using multiple t-tests with a false discovery rate cutoff value of 0.01, unpaired two-tailed t-test, Ordinary two-way ANOVA, and Pearson Correlation Coefficient using GraphPad Prism 9. Significance is designated as follows: *p < 0.05; **p < 0.01; ***p < 0.001; ****p < 0.0001; ns, not significant.

## Results

### Blocking SUMOylation suppresses the growth of KRAS-mutant cancer cells

**Fig. 1:**
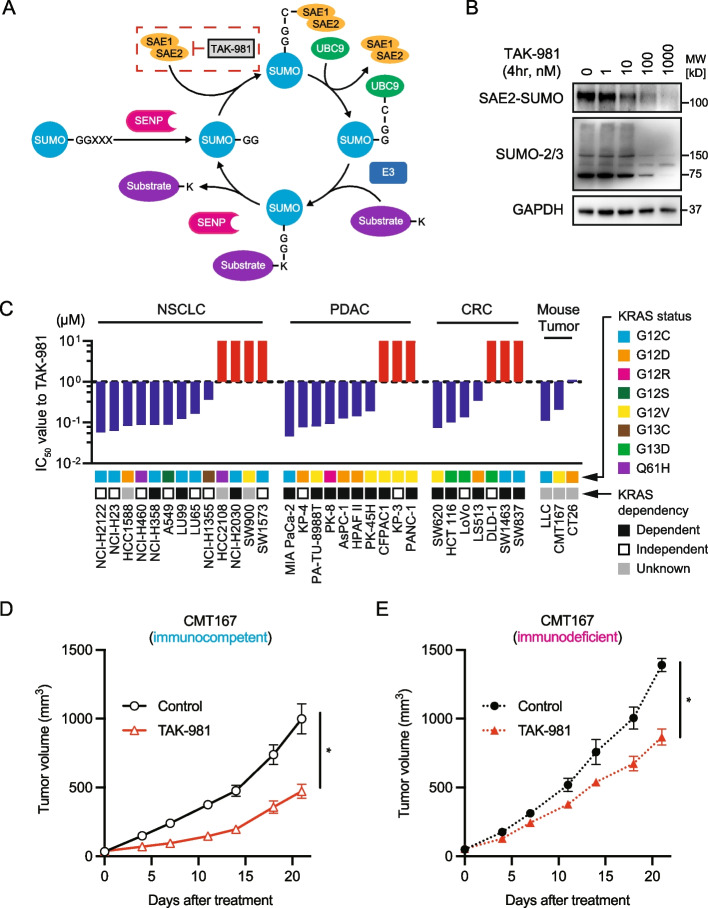
SUMOylation inhibition suppresses the growth of *KRAS*-mutant cancers. **A** The SUMOylation cycle and inhibition by TAK-981. SUMO, small ubiquitin-like modifier; SAE, SUMO-activating enzyme; UBC9, ubiquitin conjugation enzyme 9; SENP, SUMO-specific protease. **B** Immunoblotting of HCT116 cells treated with various concentrations of TAK-981 for 4 h. SAE2-SUMO conjugation was assessed using a SUMOylation assay kit and SAE2 antibody. **C** Sensitivity of human and mouse *KRAS* mutant cell lines to TAK-981. Cells were treated with nine different concentrations of TAK-981, threefold dilutions from 10 µM to 1.5 nM, or without TAK-981 for 72 h (*n =* 6 per dose). The half-maximal inhibitory concentration (IC_50_) was calculated using GraphPad Prism 9. The Y-axis represents the Y-axis of the TAK-981 IC_50_ in each cell line. The blue and red bars are visually classified as ‘sensitive’ and ‘resistant’, respectively. **D**, **E** Immunocompetent or immunodeficient mouse models using CMT167 cells. The mice were treated with vehicle (control) or TAK-981 (25 mg/kg, i.p., twice a week). Tumor volumes were plotted over time from treatment initiation (*n* ≥ 6 per group; mean ± s.e.m.). *p < 0.05 (unpaired t-test)

We first evaluated whether TAK-981 effectively blocks SAE and leads to the inhibition of SUMOylation as previously reported [9–12] (Fig. 1A). After HCT116 cells were treated with TAK-981 at various concentrations for 4 h, we observed that the drug inhibited the conjugation of SAE2 to SUMO in a dose-dependent manner and led to the deconjugation of SUMO 2/3, indicating that SUMOylation was inhibited (Fig. 1B). Next, we examined the sensitivity of TAK-981 to multiple *KRAS*-mutant human and mouse cancer cell lines using a cell viability assay. Surprisingly, regardless of the tissue type, the half-maximal inhibitory concentration (IC_50_) in almost 70% of the cell lines was less than 1 µM, which was defined as “sensitive” in this study (Fig. 1C). Moreover, sensitivity did not seem to correlate with *KRAS* mutation status or KRAS dependency, as defined by a previous report and data from the Cancer Cell Line Encyclopedia (CCLE) (Supplementary Table 3) [17]. Next, we investigated the efficacy of TAK-981 in vivo using immunocompetent and immunodeficient mouse models to determine the extent to which the antitumor response induced by SUMOylation inhibition was affected by immune-independent or immune-dependent effects. TAK-981 significantly suppressed tumor growth in both models (Fig. 1D-E, Supplementary Fig. S1), suggesting that SUMOylation inhibition exhibited an immune-independent antitumor effect.

### Correlation between sensitivity to SUMOylation inhibition and MYC expression in KRAS-mutant cancer

**Fig. 2:**
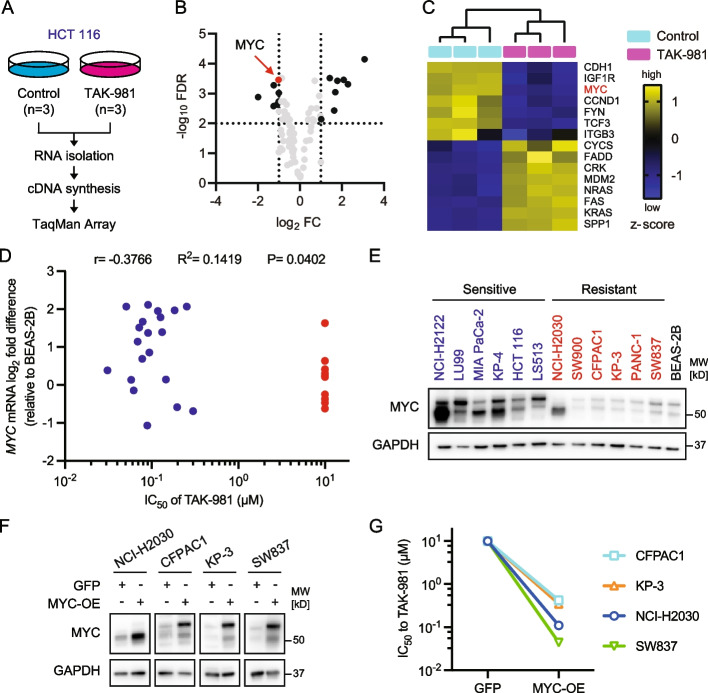
MYC expression is correlated with SUMOylation inhibition sensitivity in *KRAS*-mutant cancers. **A** Overview of the TaqMan Array analysis. HCT116 cells were treated with dimethyl sulfoxide (DMSO) or 1 µM TAK-981 for 48 h (*n =* 3). **B**, **C** Mini-volcano plot (**B**) and normalized heatmap (**C**) of differentially expressed genes (FC > I2I and FDR < 0.01, multiple t-tests) related to (**A**). FC, fold change; FDR, false discovery rate. **D** Correlation between *MYC* mRNA expression and the IC_50_ of TAK-981 (*n =* 3 for each cell line). TAK-981-sensitive cells and resistant cells are represented by blue and red dots, respectively. The IC_50_ of TAK-981-resistant cells are provisionally presented as 10 µM. r, correlation coefficient; R.^2^, square of r (Pearson correlation coefficient). **E** MYC protein expression levels determined by immunoblotting representative TAK-981-sensitive cells (blue), TAK-981-resistant cells (red), and a normal epithelial cell line (black). **F** Immunoblotting of TAK-981-resistant cells transfected with GFP or MYC vectors. GFP, Green Fluorescent Protein; MYC-OE, MYC-overexpression. **G** Change of TAK-981 sensitivity in MYC-OE cells (See also Supplementary Figure S4)

SUMOs are essential for the regulation of several cellular processes, including the cell cycle, DNA damage repair, nuclear transport, chromosomal structure, and segregation, and more than 3,600 proteins are target substrates of SUMOylation [18]. Therefore, to explore a biomarker of sensitivity to TAK-981, we investigated the change in gene expression in HCT116 cells treated with or without TAK-981 for 48 h using the TaqMan Array Human Molecular Mechanisms, a simple 92-gene assay for specific biological processes (Fig. 2A). Eight upregulated genes and seven downregulated genes among the target genes of the TaqMan Array were observed after SUMOylation inhibition (Fig. 2B-C, Supplementary Table 4–6). Among them, we focused on a downregulated *MYC* oncogene because MYC is a critical mediator of KRAS function, and MYC-driven tumorigenesis can be regulated by SUMOylation inhibition [19, 20]. Further analysis of cancer cell lines and three noncancerous cell lines as controls revealed that sensitivity to TAK-981 correlated with basal *MYC* mRNA expression levels (Fig. 2D, Supplementary Fig. S2, Supplementary Table 7). In addition, MYC protein expression levels, which can be detected at 65 kDa and/or 49 kDa by immunoblotting [21], strengthened the correlation between sensitivity to TAK-981 and *MYC* mRNA expression in representative cell lines sensitive or resistant to TAK-981 (Fig. 2E, Supplementary Fig. S3). Interestingly, TAK-981-resistant cells with MYC-low expression were sensitized to TAK-981 by MYC overexpression (Fig. 2F-G, Supplementary Fig. S4).

### Proteasomal degradation of MYC induced by SUMOylation inhibition

**Fig. 3:**
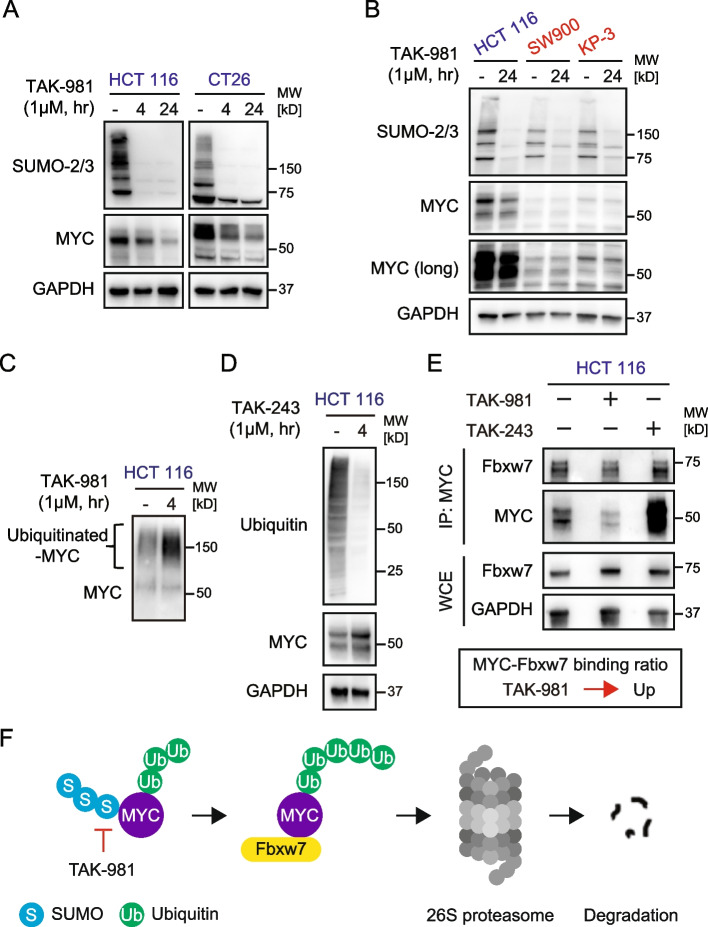
TAK-981 induces proteasomal degradation of MYC. **A**, **B** Immunoblots of cells treated with or without 1 µM TAK-981 for the indicated periods. HCT116 and CT26 cells sensitive to TAK-981, and SW900 and KP-3 cells resistant to TAK-981 are shown. **C** Immunoblotting of ubiquitinated-MYC in HCT116 cells treated with or without 1 µM TAK-981 for 4 h. Ubiquitinated-MYC was assessed using the Signal-Seeker Ubiquitination Detection kit and MYC antibody. **D** Immunoblotting of HCT116 cells treated with or without TAK-243 1 µM for 4 h. **E** Immunoblotting of HCT 116 cells treated with DMSO, TAK-981 1 µM, or TAK-243 1 µM for 4 h. Samples of upper images were immunoprecipitated with MYC. The precipitated proteins were immunoblotted with the indicated antibodies. Simultaneously, whole-cell extracts were probed with the indicated antibodies. IP, immunoprecipitation; WCE, whole-cell extract. **F** Scheme of proteasomal degradation of MYC in the SUMOylation inhibition state. TAK-981 induces a polyubiquitinated state of MYC and promotes the binding of MYC and Fbxw7, an E3 ubiquitin ligase. 26S proteasome degrades the complex, resulting in MYC downregulation. S, SUMO; Ub, ubiquitin

Next, we examined how MYC protein expression is regulated by SUMOylation inhibition. Interestingly, MYC expression levels were decreased by SUMOylation inhibition in cells sensitive to TAK-981 but not in cells resistant to TAK-981 (Fig. 3A-B). To explore the mechanism of MYC regulation by PTMs, we investigated the ubiquitination state of MYC after SUMOylation inhibition and MYC expression after ubiquitination inhibition in HCT116 cells. TAK-981 induced a highly ubiquitinated-MYC state, while a selective ubiquitination inhibitor, TAK-243, upregulated MYC expression (Fig. 3C-D). To further analyze the epigenomic regulation of MYC, MYC was immunoprecipitated after TAK-981 or TAK-243 treatment and immunoblotted with Fbxw7, an E3-ubiquitin ligase as a key player in MYC regulation [22]. Interestingly, the MYC-Fbxw7 binding ratio was increased by SUMOylation inhibition while MYC-Fbxw7 binding was decreased by ubiquitination inhibition (Fig. 3E). A short-period assay before MYC downregulation also supported that MYC bound more strongly to Fbxw7 than baseline after TAK-981 treatment (Supplementary Fig. S5). Thus, SUMOylation inhibition induced MYC downregulation via proteasomal degradation, which occured by binding MYC and Fbxw7 (Fig. 3F). Taken together, these results, suggest that SUMOylation inhibition suppresses the growth of MYC-expressing *KRAS*-mutant cancer cells.

### Enhanced antitumor effects by co-inhibition of SUMOylation and MEK

**Fig. 4:**
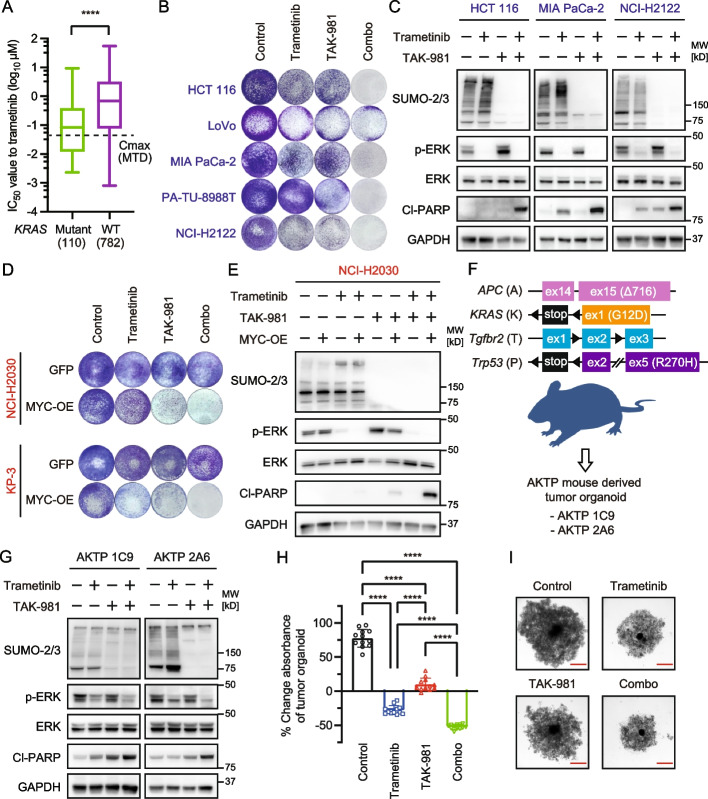
Dual inhibition of SUMOylation and MEK enhances cell growth suppression and apoptosis induction. **A** IC_50_ data for trametinib was obtained from The Genomics of Drug Sensitivity in Cancer Project. The dotted line shows the Cmax of trametinib monotherapy at the MTD. Cmax, maximum plasma concentration; MTD, maximum tolerated dose. ****p < 0.0001 (unpaired t-test). **B** Cells were stained with Diff-Quik after 3-day treatment with trametinib (30 nM), TAK-981 (1 µM), or their combination. **C** Immunoblot of cells treated 24 h after treatment with trametinib (30 nM), TAK-981 (1 µM), or their combination. **D** GFP or MYC-OE cells were stained with Diff-Quik after 3-day treatment with trametinib (30 nM), TAK-981 (1 µM), or their combination. **E** Immunoblot of NCI-H2030 GFP or MYC-OE cells treated 24 h after treatment with trametinib (30 nM), TAK-981 (1 µM), or their combination. **F** Scheme of genetically engineered AKTP mouse. AKTP 1C9 and AKTP 2A6 cells were established from GEMM tumor organoids. GEMM, genetically engineered mouse model. **G** Immunoblot of spheroid cells treated with trametinib (30 nM), TAK-981 (1 µM), or a combination of both for 24 h. **H** Spheroid growth assay. AKTP 1C9 cells were treated with trametinib (30 nM), TAK-981 (1 µM), or their combination for 5 days. Values represent the percentage change in the absorbance of the cells relative to the initial absorbance immediately before treatment (*n =* 12 per group, mean ± s.d.). ****p < 0.0001 (Ordinary two-way ANOVA). **I** Morphological evaluation of representative AKTP 1C9 cells as described in (**F**). Scale bar, 500 µm

**Fig. 5:**
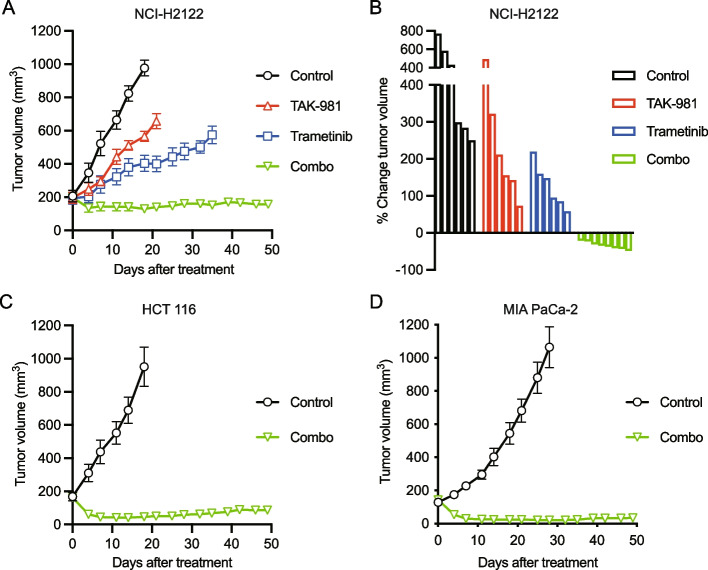
Durable remission by combination therapy with TAK-981 and trametinib in mouse models. **A** NCI-H2122 xenograft mouse model treated with vehicle (control), trametinib (0.6 mg/kg, p.o., daily), TAK-981 (25 mg/kg, i.p., twice/week), or both drugs in combination at the same dose. Tumor volumes were plotted over time from treatment initiation (*n* ≥ 6 per group; mean ± s.e.m.). **B** Waterfall plots showing the percentage change in tumor volume (relative to the initial volume) for individual NCI-H2122 tumors after 18 days of treatment. **C**, **D** Xenograft mouse models using HCT116 or MIA PaCa-2 cells treated with vehicle (control) or trametinib (0.6 mg/kg, p.o., daily) plus TAK-981 (25 mg/kg, i.p., twice a week). Tumor volumes were plotted over time from treatment initiation (*n* ≥ 6 per group; mean ± s.e.m.)

In addition, to increase the antitumor effect because the efficacy of TAK-981 monotherapy was modest in vivo, as described above, we next focused on the co-inhibition of SUMOylation and MEK because the MAPK pathway plays critical roles in *KRAS*-mutant cancer [23]. As expected, *KRAS*-mutant cancer cell lines were more sensitive to a commercially available MEK inhibitor, trametinib, than were *KRAS* wild-type cancer cell lines based on screening data from The Genomics of Drug Sensitivity in Cancer Project (GDSC) (Fig. 4A, Supplementary Table 8). Hereafter, we set the in vitro experimental concentration of trametinib to 30 nM because the maximum plasma concentration (Cmax) is less than 40 nM at the maximum tolerated dose (MTD) of monotherapy in humans [24]. Next, we examined the efficacy of combination treatment with trametinib and TAK-981 in MYC-expressing *KRAS*-mutant cancer cells. Surprisingly, this combination drastically suppressed cell growth and induced apoptosis in multiple cell lines across the different tissue types (Fig. 4B-C). Moreover, the combination was also effective in MYC-overexpressing cells that were originally resistant to TAK-981 and had low MYC-expression (Fig. 4D-E). Furthermore, we investigated whether these results could be captured in the genetically engineered mouse model (GEMM) tumor organoid-derived cells, AKTP 1C9 and 2A6 [25], which express MYC at similar levels to those of CMT167 and CT26 (Fig. 4F, Supplementary Fig. S6). These cells grew easily under spheroid culture conditions without the use of scaffolds. Interestingly, the co-inhibition of MEK and SUMOylation induced apoptosis in these GEMM tumor organoid-derived cells and significantly suppressed spheroid formation (Fig. 4G-I). These findings led us to test the efficacy of the combination of TAK-981 and trametinib in vivo using xenografted NCI-H2122 cells. All the monotherapies and combination therapy exhibited antitumor efficacy compared to that of the control, but only the combination therapy induced long-term tumor regression (Fig. 5A-B). In addition, the combination exhibited remarkable efficacy in xenografts of both HCT116 and MIA PaCa-2 cells (Fig. 5C-D).

### DNA damage accumulation induced by the combination of TAK-981 and trametinib

**Fig. 6:**
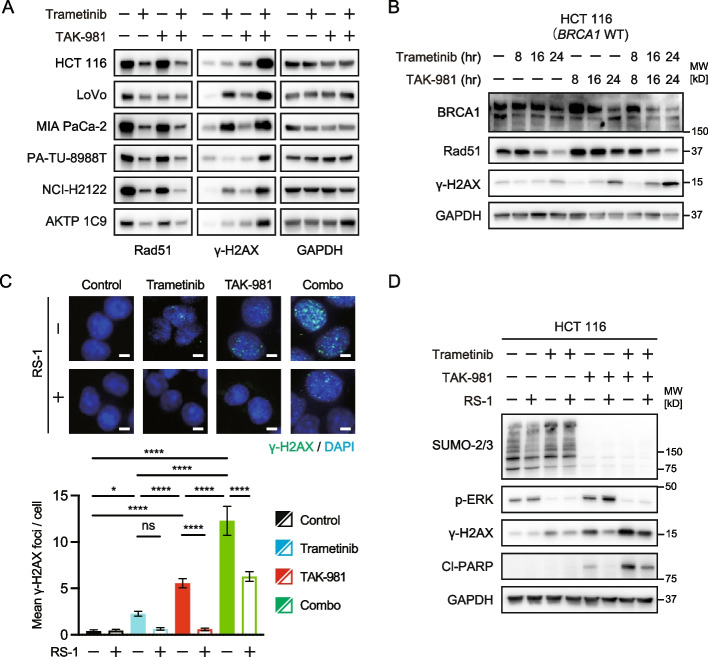
DNA damage accumulation induced by the combination of a SUMOylation inhibitor and a MEK inhibitor via Rad51/BRCA1 suppression. **A** Immunoblotting of cells treated with trametinib (30 nM), TAK-981 (1 µM), or their combination for 24 h. **B** Immunoblotting of HCT116 cells treated with trametinib (30 nM), TAK-981 (1 µM), or their combination for the indicated period. **C** Representative merged immunofluorescence images of ɣ-H2AX (green) and DAPI (blue) staining of HCT116 cells treated with the control, trametinib (30 nM), TAK-981 (1 µM), or their combination in the presence or absence of RS-1 (7.5 µM) for 24 h. The number of ɣ-H2AX foci per cell was calculated manually (100 cells per group, mean with 95% confidence interval). Scale bar, 10 μm. *p < 0.05; ****p < 0.0001; ns, not significant (Ordinary two-way ANOVA). **D** DNA damage suppression and apoptosis reduction by RS-1 in HCT 116 cells. Immunoblots of cells treated with trametinib (30 nM), TAK-981 (1 µM), or their combination in the presence or absence of RS-1 (7.5 µM) for 24 h

To explore the mechanisms underlying the effects of this combination, we investigated a part of the DNA damage repair pathway related to DNA double-strand breaks (DSBs) because MEK inhibition might induce dependence on DSB repair in *KRAS*-mutant cancer cells, and SUMOylation inhibition could block Rad51 function, which plays a critical role in DSB repair [26–29]. Surprisingly, both trametinib and TAK-981 suppressed Rad51 expression. Moreover, dual inhibition induced more robust suppression of Rad51 and cooperatively accumulated DNA damage, as indicated by ɣ-H2AX expression (Fig. 6A). In HCT116 cells, the expression of BRCA1, which is upstream of Rad51 in the DSB repair pathway, was downregulated in a time-dependent manner, especially in response to the combination treatment (Fig. 6B). In addition, RS-1, a homology-directed repair enhancer that increases the DNA-binding activity of Rad51, reduced ɣ-H2AX foci induced by treatment with trametinib, TAK-981, or the combination in HCT116 cells (Fig. 6C). We also confirmed that RS-1 suppresses DNA damage and reduces apoptosis (Fig. 6D). Taken together, these findings suggest that this combination mechanistically blocks DSB repair, resulting in the accumulation of DNA damage and the induction of apoptosis in MYC-expressing *KRAS*-mutant cancers.

## Discussion

Oncogene addiction is a characteristic of cancer cells that is dependent on cell proliferation and survival [30]. The inhibition of causal oncogenes dramatically improves the outcome of cancer patients with driver gene alterations. For instance, BCR-ABL kinase inhibitors are used for chronic myeloid leukemia and EGFR tyrosine kinase inhibitors are used for NSCLC with *EGFR* mutations. However, *KRAS*-mutant cancers are complex because of differences in *KRAS* dependency. The clinical outcomes of *KRAS*^*G12C*^-mutant NSCLC treated with KRAS^G12C^ inhibitors were worse than those of *EGFR*-mutant NSCLC treated with EGFR tyrosine kinase inhibitors [3, 4, 30]. Therefore, other treatment strategies, including epigenetic modifiers, may be useful for treating *KRAS*-mutant cancers [31].

The SUMOylation inhibitor TAK-981 is an epigenetic modifier drug with immune-modulating functions that has shown promising results in early-phase clinical trials [32, 33]. In particular, preliminary data from a phase I trial in combination with an immune checkpoint inhibitor (ICI), pembrolizumab, showed that the Cmax of TAK-981 reached more than 1 µM at the recommended phase II dose, and clinical benefits including radiographical responses were observed in multiple patients with NSCLC with a history of ICI treatment or CRC with microsatellite stability. Since the patients who responded in the clinical trials are usually resistant to ICI treatment, TAK-981 might promote antitumor responses beyond the immune response.

In this study, we demonstrated the immune-independent effects of TAK-981 and its combination with the MEK inhibitor trametinib in MYC-expressing *KRAS*-mutant cancers. While MYC expression in *KRAS*-mutant cancers was associated with sensitivity to a SUMOylation inhibitor, our limitations are that we used a biased approach to the analysis and were unable to define a cut-off value for MYC expression. However, our results are reasonable based on previous reports related to the interactions of KRAS-SUMO/SUMO-MYC/MYC-KRAS for the following reasons: 1) cell growth in *KRAS*-mutant CRC is driven by Ubc9, which is a downstream cascade in the SUMOylation pathway [7]; 2) MYC inhibition by dominant-negative *MYC* mutation eradicates *KRAS*-mutant GEMM [34]; and 3) hyperactivation of MYC is associated with sensitivity to pharmacological SUMO inhibition in PDAC [35]. In addition, it would be important that MYC-overexpression increased the sensitivity to SUMOylation inhibition in TAK-981-resistant cells. Although the mechanism by which MYC can be regulated by SUMOylation and ubiquitination is unknown [36], one hypothesis regarding MYC downregulation by TAK-981 is that proteasomes can degrade residual ubiquitinated MYC by blocking SUMOylation because SUMOylation and ubiquitination might function competitively depending on the substrate. Here, we revealed how MYC is regulated by the competitive balance between SUMOylation and ubiquitination, although further epigenetic studies on MYC and unbiased approaches such as mass spectrometry and RNA-seq are warranted. Moreover, in *KRAS*-mutant human cell lines used in this study, *TP53* mutations or null were detected in 90% of TAK-981-resistant cells (9 out of 10) and 60% of TAK-981-sensitive cells (12 out 20). Therefore, *TP53* function may affect the resistance mechanisms to SUMOylation inhibition in *KRAS*-mutant cancers.

We found that DNA damage accumulated via Rad51/BRCA1 inhibition by both TAK-981 and trametinib, particularly in combination. These new findings explain why dual inhibition of SUMOylation and MEK induced dramatic antitumor responses in MYC-expressing *KRAS*-mutant cancer cells in vitro and in vivo without immune reactions. However, the causes of regulation of Rad51 expression and the factors that distinguish the induction of DNA damage accumulation and apoptosis remain unclear. This may vary according to the cancer type or DNA damage response/repair gene alterations.

## Conclusion

In this study, we showed that dual inhibition of SUMOylation and MEK could conquer MYC-expressing *KRAS*-mutant cancers by complementarily enhancing DNA damage accumulation. Our results provide a strategy for treating *KRAS*-mutant cancers through personalized selection based on MYC expression.

### Supplementary Information

Supplementary Material 1: Supplementary Figure S1. Individual tumor volume plot of the CMT167 model. Supplementary Figure S2. Correlation between MYC mRNA expression and the IC50 of TAK-981. Supplementary Figure S3. MYC protein expression levels determined by immunoblotting in representative TAK-981-sensitive cells (blue) and resistant cells (red). Supplementary Figure S4. Change of SUMOylation inhibition sensitivity by MYC overexpression in TAK-981-resistant cells. Supplementary Figure S5. SUMOylation inhibition of short period in HCT 116 cells. Supplementary Figure S6. MYC expression in AKTP cells.

Supplementary Material 2: Supplementary Table 1. Cell line information. Supplementary Table 2. List of antibodies. Supplementary Table 3. KRAS dependency data. Supplementary Table 4. DeltaCT values of target genes normalized to GAPDH in HCT116 cells treated with or without TAK-981. Supplementary Table 5. Fold-change in target gene expression after TAK-981 treatment in HCT 116 cells. Supplementary Table 6. Normalized Z-scores of significant DEGs in HCT116 cells treated with TAK-981. Supplementary Table 7. *MYC* mRNA expression compared to that in normal cell lines. Supplementary Table 8. Trametinib sensitivity data from the GDSC.

## Data Availability

All data supporting the findings of this study are available from the lead contact author upon reasonable request.

## Abbreviations

BCR-ABL: Breakpoint cluster region-Abelson
BRCA1: Breast cancer susceptibility gene1
CCLE: Cancer Cell Line Encyclopedia
Cmax: Maximum plasma concentration
CRC: Colorectal cancer
DSB: DNA double-strand break
EGFR: Epidermal growth factor receptor
GDSC: The Genomics of Drug Sensitivity in Cancer Project
GEMM: Genetically engineered mouse model
GFP: Green Fluorescent Protein
ɣ-H2AX: Gamma-histone 2A family member X
IC50: Half-maximal inhibitory concentration
ICI: Immune checkpoint inhibitor
KRAS: V-Ki-ras2 Kirsten rat sarcoma 2 viral oncogene homolog
MAPK: Mitogen-activated protein kinase
MTD: Maximum tolerated dose
MYC: V-myc avian myelocytomatosis viral oncogene homolog
NSCLC: Non-small cell lung cancer
PDAC: Pancreatic ductal adenocarcinoma
PTM: Post-translational modification
Rad51: Recombinant DNA repair protein 51
SAE: Small ubiquitin-like modifier-activating enzyme
SENP: Small ubiquitin-like modifier-specific protease
SUMO: Small ubiquitin-like modifier
UBC9: Ubiquitin-conjugating enzyme 9
