# Supplementary material for: Dual inhibition of SUMOylation and MEK conquers MYC-expressing KRAS-mutant cancers by accumulating DNA damage
Source: J Biomed Sci. 2024 Jul 11;31:68. doi: 10.1186/s12929-024-01060-3 (PMC11238369; doi:10.1186/s12929-024-01060-3)
Supplement: Supplementary file 1 — Supplementary Material 1: Supplementary Figure S1. Individual tumor volume plot of the CMT167 model. Supplementary Figure S2. Correlation between MYC mRNA expression and the IC50 of TAK-981. Supplementary Figure S3. MYC protein expression levels determined by immunoblotting in representative TAK-981-sensitive cells (blue) and resistant cells (red). Supplementary Figure S4. Change of SUMOylation inhibition sensitivity by MYC overexpression in TAK-981-resistant cells. Supplementary Figure S5. SUMOylation inhibition of short period in HCT 116 cells. Supplementary Figure S6. MYC expression in AKTP cells. [file 12929_2024_1060_MOESM1_ESM.pdf]

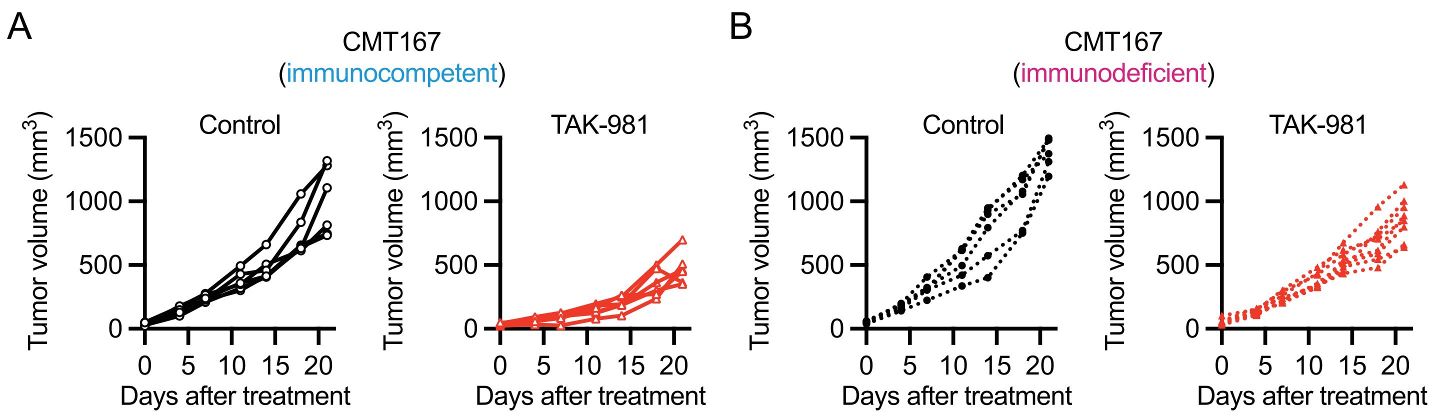

Supplementary Figure S1. Individual tumor volume plot of the CMT167 model.
Individual tumor volumes related to Fig. 1D and 1E were plotted in Supplementary Fig. 1A and 1B, respectively.

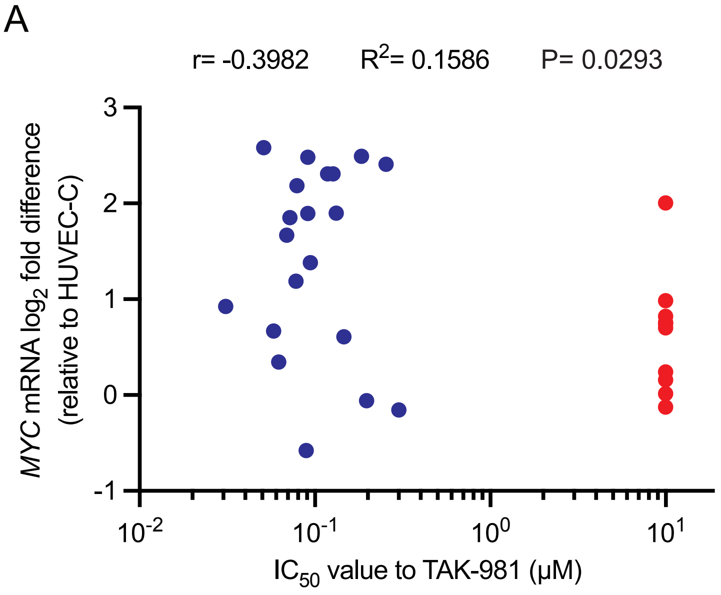

Supplementary Figure S2. Correlation between MYC mRNA comparative expression and IC50 value to TAK-981. TAK-981 sensitive cells and resistant cells were represented by blue dots and red dots, respectively. IC50 values of TAK-981 resistant cells were provisionally presented as 10 μM. r, correlation coefficient; R2, square of r (Pearson correlation coefficient).

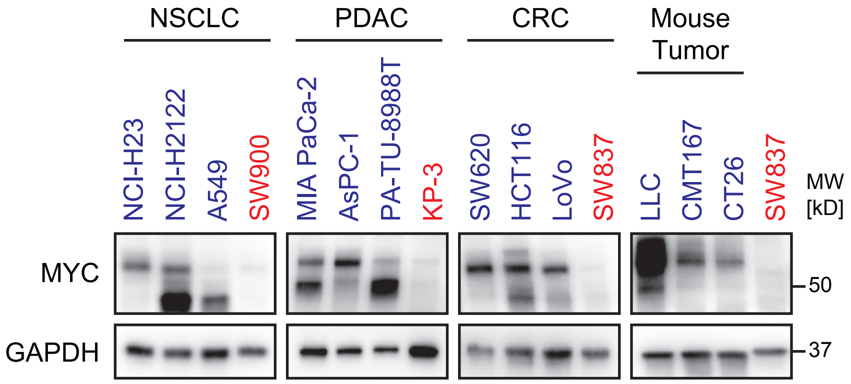

Supplementary Figure S3. MYC protein expression levels by immunoblot of representative TAK-981 sensitive cells (blue) and resistant cells (red). NSCLC, non-small cell lung cancer; PDAC, pancreatic ductal adenocarcinoma; CRC, colorectal carcinoma.

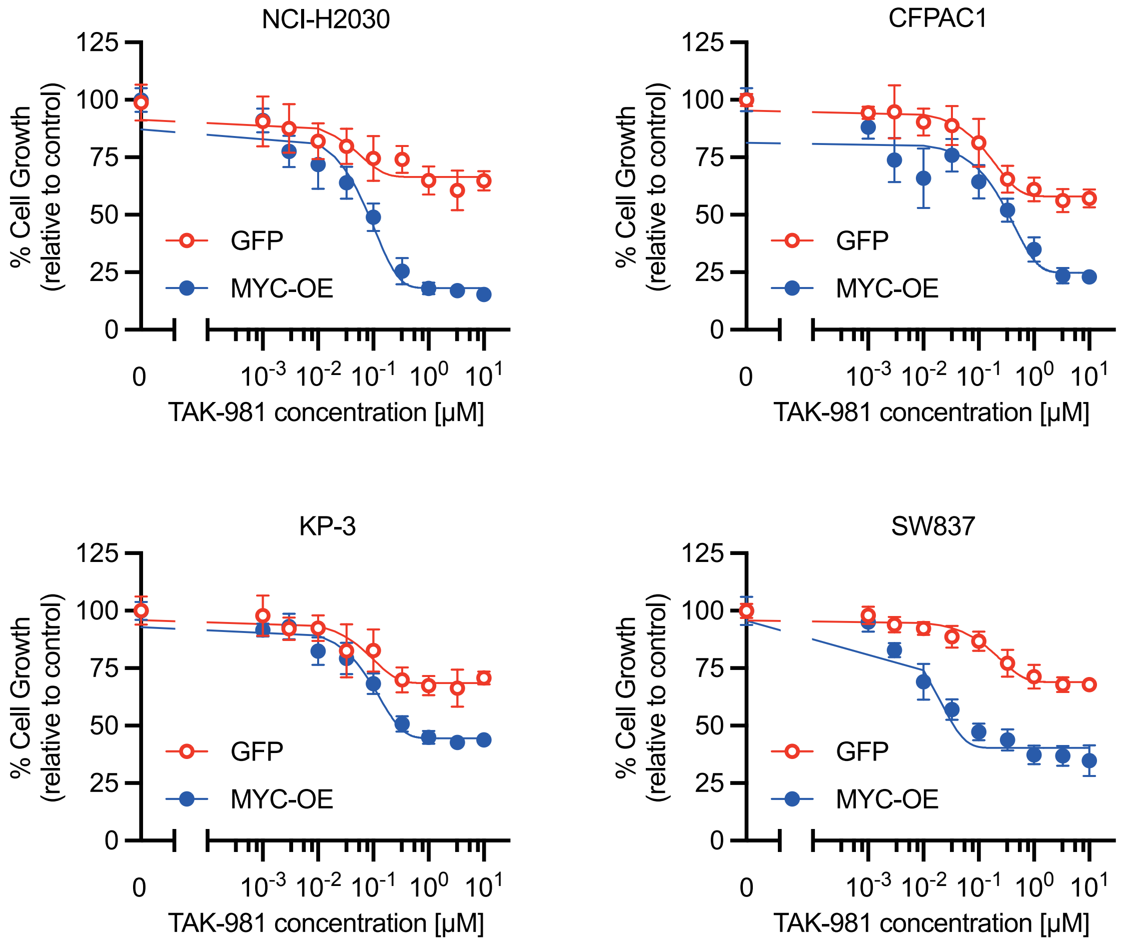

Supplementary Figure S4. Change of SUMOylation inhibition sensitivity by MYC overexpression in TAK-981-resistant cells. GFP, Green Fluorescent Protein; MYC-OE, MYC-overexpression.

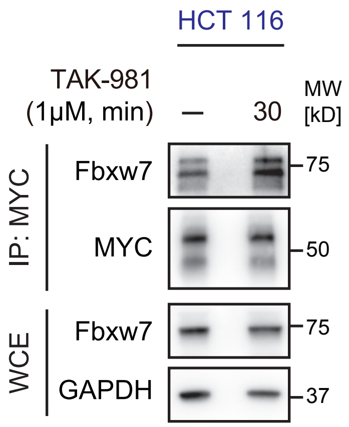

Supplementary Figure S5. SUMOylation inhibition of short-period in HCT 116 cells.
HCT 116 cells were treated with DMSO or 1 µM TAK-981 for 30 minutes. Samples were anlyzed as shown in Figure 3E. IP, immunoprecipitation; WCE, whole cell extract.

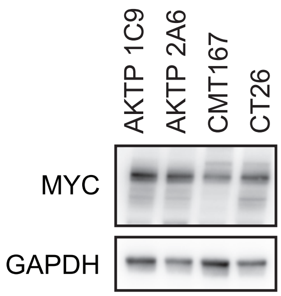

Supplementary Figure S6. MYC expression in AKTP cells.
Immunoblot of indicated cells.
